# Knowledge of *Chlamydia trachomatis *among men and women approached to participate in community-based screening, Scotland, UK

**DOI:** 10.1186/1471-2458-10-794

**Published:** 2010-12-30

**Authors:** Karen Lorimer, Graham J Hart

**Affiliations:** 1 Institute for Applied Health Research, Glasgow Caledonian University, Glasgow, UK; 2 Division of Population Health, University College London, London, UK

## Abstract

**Background:**

Poor awareness and knowledge of *Chlamydia trachomatis *could be a barrier to uptake of screening. This study aimed to determine the level of awareness and knowledge of chlamydia among young people who were being approached in a variety of community settings and offered opportunistic screening.

**Methods:**

Men and women aged 16-24 years were approached in education, health and fitness, and workplace settings and invited to complete a self-administered questionnaire then provide a urine sample for chlamydia testing. Follow-up semi-structured interviews with 24 respondents were carried out after test results were received.

**Results:**

363 questionnaires were completed (43.5% from men). Whilst awareness of chlamydia was high, knowledge decreased as questions became increasingly focussed so that around half of respondents were unaware of the asymptomatic nature of chlamydia infections. Men's knowledge of symptoms was consistently lower than women's, with most men failing to identify unusual discharge as a symptom in men (men 58.3%, female 45.8%, p = 0.019)*; *fewer men knew unusual discharge was a symptom among women (men 65.3% female 21.4%, p < 0.001). The asymptomatic nature of the infection resonated with respondents and was the commonest piece of information they picked up from their participation in the study.

**Conclusions:**

Despite scientific gains in understanding chlamydia infection, public understanding remains limited. Greater efforts are required to translate scientific evidence to the public. An improvement in knowledge may maximise gains from interventions to improve detection.

## Background

*Chlamydia trachomatis *is the most common bacterial sexually transmitted infection in the UK [1], with a high estimated population prevalence of 3%, rising to around 10% among young people aged under 25 years [2-4], and high transmission potential [5,6]. In 2008 there were 83, 214 diagnoses of chlamydia in the UK (among those aged under 25 years), a slight increase from 81, 652 in 2007[7].

The opportunistic approach to screening, as recommended by screening guidelines for Scotland [3] and as undertaken in England as part of the National Chlamydia Screening Programme (NCSP) has been criticised as 'unlikely to be sufficient to control the spread of chlamydia' due to 'low annual coverage and infrequent screening' [8]. The most recent data for the English NCSP revealed an overall coverage of 15.9% among 15-24 year olds between April 2009 -December 2009 [9], which is lower than 30-40% effective screening rate among those aged under 25 years estimated in a National Institute for Health and Clinical Excellence (NICE) rapid review as necessary to effect a reduction in prevalence [10,11]. This failure to reach young people is occurring despite the introduction of non-invasive tests, which has created new opportunities for screening in a variety of non-medical settings, such as: student bars [12]; in field settings (such as parks, street corners and areas where youth congregate)[13]; mobile clinics [14]; schools and universities [15-17]; health and fitness and workplace settings [18]; as well as enabling postal testing from community pharmacies [19] and in the home setting [20-22]. One reason for this might be that despite scientific gains in understanding chlamydia infection, public understanding of chlamydia remains limited. Qualitative studies have reported: some young people think they can die from having chlamydia [23]; belief that it is a 'woman's disease' [24,25]; belief that the test is painful and invasive [24,26]; and perceptions that only 'slappers' and 'dirty' people contract chlamydia [24,26-29].

Poor knowledge could obstruct the effectiveness of any screening approach, as young people are unlikely to take up the offer of screening if they are unaware of key clinical features, such as the largely asymptomatic nature of the infection and associated sequelae. It is therefore important to continue to assess and monitor levels of awareness and knowledge of chlamydia so as to better communicate messages about chlamydia to young people. Where there are no national screening programmes (such as in Scotland) better ways to inform young people will be required. This study aimed to determine the level of awareness and knowledge of chlamydia among young people who were approached in a variety of community settings in Glasgow, Scotland and offered opportunistic screening, by urine sample. The willingness of young people to accept the offer of screening and provide a urine sample is reported elsewhere [18,26]; this paper reports the knowledge of those respondents.

## Methods

Young people aged 16-24 years were approached in a large further education college [in the UK these vocational institutions are a level above compulsory education but below university], health and fitness (local authority-run facilities rather than private gyms), and workplace settings (two call centres - office environments that provided non-specialist telephone-based consumer services for energy companies) and invited to take part in a chlamydia screening study. A convenience sample was employed, due to the way in which young people used the settings which prohibited a more systematic approach (further detail is provided elsewhere [18]).

The study took place over the course of 4 weeks in each setting (between October 2004 - April 2005). One week prior to, and throughout, the study, posters placed around the study venues provided information about chlamydia. Young people who appeared age-eligible were approached: in the main canteen in the education setting; in the main foyers of the health and fitness settings; and, in a kitchen/'chill-out' area of the two contact centres. The lead author (KL) approached young people, established their age for eligibility, explained the study to them and informed them about chlamydia (verbally, and by giving them a study leaflet - see Additional file 1 study leaflet). The leaflet provided information on what chlamydia is, possible male and female symptoms and that the test can be performed on a sample of urine. Willing participants provided written consent and were requested to complete a self-administered questionnaire, which asked for their knowledge of chlamydia and their views on screening. Participants were to complete the questionnaire immediately in the location (e.g. at their table in the canteen) and return it to the researcher. In practice a few participants in the workplace setting took the questionnaire away and returned it within an hour or two. Privacy levels for participants completing a questionnaire varied across the settings, with large numbers of people using the education setting canteen area compared to the commonly quiet location of the health and fitness main foyers.

Upon returning the questionnaire participants were invited to provide a urine sample for chlamydia testing - they could decline and remain in the study. Throughout fieldwork, a record of the number of people approached was made, as well as general observations of young people's non-verbal response to the offer of chlamydia screening being noted in fieldwork diaries (e.g. young women hiding their samples in jacket pockets).

The questionnaire (see Additional file 2 questionnaire) captured respondents' knowledge of chlamydia, views towards screening, sexual behaviour and willingness to provide a urine sample in the setting. The knowledge section of the questionnaire contained 17 items. Four questions asked how infection occurs, symptoms associated with chlamydia infection in women and symptoms among men (choosing from a list of symptoms), and testing method. From eleven statements to which respondents could answer 'True', 'False' or 'Not sure' (table 1), a mean knowledge score was calculated (one point for a correct answer, 0 for an incorrect answer, answering not sure or providing no answer). The maximum score achievable was 11. Respondents were also asked to respond to two statements: 'I'd know if I had chlamydia' and 'I'd only think about chlamydia if I had symptoms' (responding to a 5-point Likert scale from Strongly Agree to Strongly Disagree).

**Table 1:** True/False questions used to assess knowledge of chlamydia

		*Correct response (%)*	*Incorrect response* *(%)*	*Answered 'Not sure'* *(%)*	*P Value*	*Unadjusted OR* *(95% CI)*
1. You can catch chlamydia from toilet seats (F)	Male	110 (70.5)	14 (9.0)	32 (20.5)		1
	Female	165 (83.8)	9 (4.6)	23 (11.7)	**0.012**	1.80 (1.11-2.92)
2. Men with chlamydia might not have symptoms (T)	Male	100 (63.7)	19 (12.1)	38 (24.2)		1
	Female	148 (74.4)	23 (11.6)	28 (14.1)	0.082	1.50 (0.96-2.35)
3. Most women will not develop symptoms of chlamydia (T)	Male	39 (25.7)	51 (33.6)	62 (40.8)		1
	Female	123 (62.4)	31 (15.7)	43 (21.8)	**<0.001**	4.57 (2.89-7.22)
4. Only women get chlamydia (F)	Male	149 (95.5)	0	7 (4.5)		1
	Female	188 (94.0)	1 (0.5)	11 (5.5)	0.613	0.66 (0.29-1.54)
5. Chlamydia can affect men's fertility (T)	Male	113 (72.4)	6 (3.8)	37 (23.7)		1
	Female	123 (61.5)	9 (4.5)	68 (34.0)	0.090	1.16 (0.40-3.33)
6. Chlamydia can affect women's fertility (T)	Male	121 (78.6)	2 (1.3)	31 (20.1)		1
	Female	174 (87.0)	5 (2.5)	21 (10.5)	0.064	1.95 (0.37-10.1)
7. Chlamydia can cause eye infections (conjunctivitis) (T)	Male	18 (11.5)	64 (41.0)	74 (47.4)		1
	Female	36 (18.1)	89 (44.7)	74 (37.2)	0.084	1.12 (0.74-1.71)
8. Once you get chlamydia you can' t get rid of it (F)	Male	110 (71.0)	19 (12.3)	26 (16.8)		1
	Female	169 (84.5)	17 (8.5)	14 (7.0)	**0.005**	2.04 (1.24-3.35)
9. You can get chlamydia more than once (T)	Male	92 (59.4)	17 (11.0)	46 (29.7)		1
	Female	166 (83.0)	4 (2.0)	30 (15.0)	**<0.001**	3.05 (1.90-4.88)
10. Women's smear tests would detect chlamydia (F)	Male	16 (10.4)	52 (33.8)	86 (55.8)		1
	Female	50 (25.0)	92 (46.0)	58 (29.0)	**<0.001**	2.86 (1.56-5.25)
11. 'The Pill' prevents sexual infections (F)	Male	127 (81.9)	5 (3.2)	23 (14.8)		1
	Female	193 (96.5)	1 (0.5)	6 (3.0)	**<0.001**	3.92 (1.94-7.92)

Correct answer in parentheses.

A sub-sample of participants who completed a questionnaire and consented to take part in a follow-up interview was contacted by telephone after test results had been sent (around 1-2 weeks post test). The interviews were designed to explore further their knowledge of chlamydia, sexual lifestyles, views towards community-based screening as well as reasons for accepting or declining the offer of screening in a community setting. Regarding knowledge, respondents were asked to describe their awareness and knowledge of chlamydia (including symptoms and consequences) and from where they obtained this information (see Additional file 3 topic guide). All interviews were conducted either in a private room at the university or in the study setting, and were between 30 and 90 minutes in duration. All participants who were willing to take part in a follow-up interview were interviewed (n = 24; 18 had provided a sample for testing and 6 had refused).

Analysis of differences in proportions was carried out using the χ^2 ^test, with Fisher Exact test used where expected cell counts is less than 5. Univariate logistic regression tested the association between knowledge and gender. Respondents' ages were categorised bi-modally (16-19 years; 20-24 years). Interviews were recorded and transcribed verbatim before being coded using Atlas.ti (Scientific Software Development, Berlin, Germany). Transcripts were read repeatedly for emerging and recurrent themes. Respondents' knowledge became a focus of analysis. Due to the gendered response when approaching respondents in the study settings, and the gender differences in knowledge that emerged from analysis of questionnaire data, the qualitative data were examined for gender differences in knowledge. The recurring themes identified by KL were discussed and refined with GH. Ethics approval was granted by the University of Glasgow Faculty of Medicine Ethics Committee.

## Results

A total of 363 (84%) questionnaires were completed from all those approached: 158 (43.5%) from men and 205 (56.5%) from women; by setting, 126 (34.7%) from education, 133 (36.6%) from health and fitness, and 104 (28.6%) from workplace. Twenty four respondents later participated in a follow-up semi-structured interview (10 women, 14 men). By setting, 5 participated from the education setting, 9 from health and fitness, and 10 from workplace.

The mean age of all participants was 20 years, with more 16 to 19 year olds recruited from the education setting (53.7%) than health and fitness (29.3%) or workplace settings (23.1%).

### Questionnaire responses

#### Awareness

The majority (93%) of respondents had heard of chlamydia prior taking part in the study, with no significant gender differences. Setting was significantly associated with respondents having previously heard of chlamydia, with education and workplace setting respondents more likely to have heard of chlamydia than health and fitness respondents (χ^2 ^test, *p *= 0.004). All respondents, except one, correctly identified chlamydia as being a sexually transmitted infection (99.2%). The majority (99.4%) of all study respondents correctly identified unprotected sex (no condom) as the primary means of sexual transmission. The majority of survey respondents (97.2%) knew chlamydia could be tested using a urine sample.

#### Knowledge of the clinical features of chlamydia

There were a number of misconceptions in relation to symptoms of chlamydia infection (tables 2, 3, 4, with correct symptoms in bold), with variation in level of misconception between symptoms. For symptoms of chlamydia in women, no respondent believed dizziness was a symptom whereas 80.2% failed to correctly identify 'Pain in lower stomach' as a possible symptom. By gender (table 2), significantly fewer women answered incorrectly than men to: unusual discharge, pain or stinging when urinating, pain during sex and pain in lower stomach.

**Table 2:** Number and percentage of respondents who answered incorrectly to various symptoms of chlamydia trachomatis infection in women and men, by gender

*Symptoms in women*	*Male* *N (%)*	*Female* *N (%)*	*Total* *N (%)*	**χ**^**2**^***test*** *p*
**Unusual discharge**	96 (65.3)	43 (21.4)	139 (39.9)	**<0.001**
**Pain or stinging when urinating**	89 (60.5)	84 (41.8)	173 (49.7)	**0.001**
Dizziness	0	0	0	**-**
Headache	0	5 (2.5)	5 (1.4)	0.054
**Pain during sex**	100 (68.0)	95 (47.3)	195 (56.0)	**<0.001**
**Pain in lower stomach**	133 (90.5)	146 (72.6)	279 (80.2)	**<0.001**
**Itch and/or rash**	125 (85.0)	154 (76.6)	279 (80.2)	0.052

***Symptoms in men***				

**Unusual discharge from tip of penis**	91 (58.3)	92 (45.8)	183 (51.3)	**0.019**
**Pain and/or burning when urinating**	71 (45.5)	92 (45.8)	163 (45.7)	0.961
Dizziness	0	0	0	-
Headache	0	2 (1.0)	2 (0.6)	0.212
**Pain/swelling in testicles**	117 (75.0)	150 (74.6)	267 (74.8)	0.936
Itchiness around groin area	19 (12.2)	31 (15.4)	50 (14.0)	0.381
Rash	19 (12.2)	26 (12.9)	45 (12.6)	0.831

Correct symptoms are indicated by bold typeface.

Significant relationships between gender and knowledge of symptoms are indicated by bold p-values.

**Table 3:** Number and percentage of respondents who answered incorrectly to various symptoms of chlamydia trachomatis infection in women and men, by study setting

*Symptoms in women*	*Education* *N (%)*	*Health & fitness* *N (%)*	*Workplace* *N (%)*	*Total* *N (%)*	**χ**^**2**^***test*** *p*
**Unusual discharge**	44 (37.3)	65 (49.2)	30 (30.6)	139 (39.9)	**0.013**
**Pain or stinging when urinating**	59 (50.0)	73 (55.3)	41 (41.8)	173 (49.7)	0.130
Dizziness	0	0	0	0	**-**
Headache	1 (0.8)	1 (0.8)	3 (3.1)	5 (1.4)	0.280
**Pain during sex**	64 (54.2)	78 (59.1)	53 (54.1)	195 (56.0)	0.668
**Pain in lower stomach**	100 (84.7)	106 (80.3)	73 (80.2)	279 (80.2)	0.170
**Itch and/or rash**	95 (80.5)	118 (89.4)	66 (67.3)	279 (80.2)	**<0.001**

***Symptoms in men***					

**Unusual discharge from tip of penis**	62 (50.8)	77 (58.3)	44 (42.7)	183 (51.3)	0.059
**Pain and/or burning when urinating**	58 (47.5)	67 (50.8)	38 (36.9)	163 (45.7)	0.093
Dizziness	0	0	0	0	-
Headache	1 (0.8)	1 (0.8)	0	2 (0.6)	0.664
**Pain/swelling in testicles**	87 (71.3)	101 (76.5)	79 (76.7)	267 (74.8)	0.551
Itchiness around groin area	17 (13.9)	17 (12.9)	16 (15.5)	50 (14.0)	0.844
Rash	16 (13.1)	11 (8.3)	18 (17.5)	45 (12.6)	0.109

Correct symptoms are indicated by bold typeface.

Significant relationships between gender and knowledge of symptoms are indicated by bold p-values.

**Table 4:** Number and percentage of respondents who answered incorrectly to various symptoms of chlamydia trachomatis infection in women and men, by age group

*Symptoms in women*	*16-19 years* *N (%)*	*20-24 years* *N (%)*	*Total* *N (%)*	**χ**^**2**^***test*** *p*
**Unusual discharge**	57 (44.5)	82 (37.6)	139 (40.2)	0.205
**Pain or stinging when urinating**	73 (57.0)	100 (45.9)	173 (50.0)	**0.045**
Dizziness	0	0	0	-
Headache	5 (3.9)	0	5 (1.4)	**0.003**
**Pain during sex**	87 (68.0)	108 (49.5)	195 (56.4)	**0.001**
**Pain in lower stomach**	118 (92.2)	159 (72.9)	277 (80.1)	**<0.001**
**Itch and/or rash**	106 (82.8)	171 (78.4)	277 (80.1)	0.326

***Symptoms in men***				

**Unusual discharge from tip of penis**	75 (58.6)	107 (47.3)	182 (51.4)	**0.042**
**Pain and/or burning when urinating**	68 (53.1)	95 (42.0)	163 (46.0)	**0.044**
Dizziness	0	0	0	-
Headache	2 (1.6)	0	2 (0.6)	0.059
**Pain/swelling in testicles**	106 (82.8)	158 (69.9)	264 (74.6)	**0.007**
Itchiness around groin area	20 (15.6)	30 (13.3)	50 (14.1)	0.542
Rash	10 (7.8)	34 (15.0)	44 (12.4)	**0.048**

Correct symptoms are indicated by bold typeface. Significant relationships between age group and knowledge of symptoms are indicated by bold p-values.

For symptoms of chlamydia in men, a similar variation in numbers answering incorrectly emerged, with no respondent identifying dizziness as a symptom but 74.8% failing to identify pain/swelling in testicles as a possible symptom (table 2). Significantly (χ^2 ^*test p = 0.019*) more men than women incorrectly responded to 'unusual discharge' as a symptom among men.

By setting (table 3) there was little variation in incorrect responses. Significantly fewer workplace respondents answered incorrectly to unusual discharge and itch and/or rash symptoms in women compared with education and health and fitness respondents. Whilst there was little variation in incorrect responses between settings, when comparing responses by age groups (16-19; 20-24 years) older respondents provided significantly fewer incorrect responses to four of the symptoms in women and three of the symptoms in men (table 4).

The overall mean score for the eleven 'true/false' statements was 6.9 (range 0-11). Women had a significantly higher knowledge than men (mean score 7.4 compared with 6.2, respectively; χ^2 ^test *p *< 0.001). Table 1 shows that respondents were more sure of some true/false statements than others, such as knowing that both sexes can acquire chlamydia (statement 4, women 94.0% men 95.5%) and that 'The Pill' does not prevent STIs (statement 11, women 96.5% men 81.9%). In contrast, respondents were less sure of chlamydia causing conjunctivitis (statement 7) with 18.1% of women and 11.5% of men answering correctly; and that women's smear test do not test for chlamydia (statement 10), with 25.0% of women and 10.4% of men answering correctly. Men provided more uncertain responses than women to 9 of the 11 statements. Significantly more women than men answered correctly to statement 1 (you can catch chlamydia from toilet seats, 83.8% *v *70.5%, p = 0.012), statement 3 (most women will not develop symptoms of chlamydia, 62.4% *v *25.7%, p < 0.001), statement 8 (once you get chlamydia you can't get rid of it, 84.5% *v *71.0% p = 0.005), statement 9 (you can get chlamydia more than once, 83.0% *v *59.4% p < 0.001), statement 10 (women's smear tests would detect chlamydia, 25.0% *v *10.4% p < 0.001) and statement 11 ('The Pill' prevents sexual infections, 96.5% *v *81.9% p < 0.001) (table 1). Men's knowledge was higher on statements 4 and 5, but this did not reach significance.

More respondents disagreed (47.4%) with the statement 'I'd know if I had chlamydia' than agreed (14.9%), but around 1 in 3 were not sure (36.4%). There were no differences by gender, setting or age group. Respondents were also asked to respond to the statement 'I'd only think about chlamydia if I had symptoms': most (45.5%) disagreed, whilst a third (36.6%) agreed and 17.9% were not sure. Men were significantly more likely to agree to this statement (45.6% of men compared with 29.8% of women, χ^2 ^test, *p *= 0.006).

### Interview and participant observation data

#### Awareness of chlamydia

The questionnaire data revealed that the majority (93%) of respondents had heard of chlamydia prior to taking part in this study. However, in each of the settings, when women were approached it was common for them to respond confidently and with certainty when asked if they had heard of chlamydia before, for example, "Oh, yes, uh huh, I've heard of it" and "Chlamydia yes I've heard of it.." (Fieldnotes, Workplace, April 2005). In contrast, men were less certain about having heard of chlamydia, with the researcher often receiving blank looks and being required to provide more explanation to men than to the women about chlamydia before they affirmed their awareness. During interviews, the survey findings and observations were borne out: all interviewees described being aware of chlamydia prior to the posters about the study going up in the setting. What emerged across the interviews was the superficiality of their self-reported knowledge, with some conveying only having heard of it.

"I'd heard of chlamydia but I didn't know what it was, really..."

(Interviewee #75, Male, Age 17, Education).

#### Knowledge of chlamydia

When asked in more detail about their knowledge, most interviewees spoke of their knowledge in tentative vague terms:

*I: Tell me what you know about chlamydia*.

*R: I don't know a lot about it to be honest [pause]. See, I've heard about it, I don't really know, obviously, all the ins and outs of it, but I know what it is, as much as I kinda, I think I know kind of thing, but em...I don't know a lot about it*.

(Interviewee #309, Female, Age 20, Workplace).

Only two interviewees had more detailed knowledge about chlamydia: one had worked in a laboratory which tested samples for STIs and the other had experience of working on a sexual health advice line. Both interviewees understood the largely asymptomatic nature of chlamydia and the possible effects on fertility.

Interviewees frequently contextualised their own poor knowledge of chlamydia by reference to the absence of knowledge regarding this topic on the part of young people in society generally. The young woman quoted above offered the following comment immediately after discussing her own perceived low knowledge of chlamydia:

"...but I don't think many, like most people I know, I don't think they know all about this stuff so, I mean they're about my age ...I dunno...I just think it's, there's just hardly any information about this and I think there should be."

(Interviewee #309, Female, Age 20, Workplace).

Although few interviewees could describe possible sequelae, more women than men spoke of knowing the potential fertility problems women could have following chlamydial infection.

"I didn't know any side effects or anything, apart from infertility...it was the only one I knew, and I didn't know what symptoms there was or anything like that."

[sic] (Interviewee #255, Female, Age 22, Health & Fitness).

Most interviewees, particularly the men, spoke of being surprised to learn that the majority of people would have no sign of symptoms of chlamydia infection.

"I was quite shocked by that, cos I always thought, well for most things like that, that you'd know, y'know. I always thought I would know, I'd have some sort of symptom, so yeah, I was really surprised when I read that most people don't always know."

(Interviewee #237, Male, Age 24, Health & Fitness)

The asymptomatic nature of the infection was the commonest piece of information interviewees reported as having learned from taking part in the study, and indeed, as reported elsewhere [26], was among the reasons for many being willing to provide a sample for testing as part of the study:

R: I'd heard of chlamydia before I spoke to you but I didn't know that you might not know you have it. I'd always thought that with things like this you'd know, like you'd get a discharge or something would just be like funny down there [laughs] but em, yeah I know that that's not always the case now and also that it can cause like problems, women not getting pregnant and stuff, I didn't really know that

KL: You say you know those things from taking part in this study?

*R: Yeah, like you don't always know or as you said 'you can't tell by looking'. I remember you saying that to me*.

(Interviewee #364, Male, Age 23, Workplace).

Most interviewees spoke about their own experience of sex education and offered views on the quality of sex education more generally. A typical view from both male and female interviewees was that their low knowledge about chlamydia was the 'fault' of their poor sex education. A few couched their statements in normative terms:

KL: what do you remember being taught about sexual infections in sex education?

*R: No much. I remember it was like AIDS and stuff but that's really all, HIV, that's all I kind of remember. I think we really should have been taught about this stuff, like see the first time really that I've ever went into anything in any detail has been with yourself*.

(Interviewee #308, Male, Age 22, Workplace).

The most common STI interviewees remembered being discussed during their sex education was HIV/AIDS. Most interviewees commented that they did not remember being taught about chlamydia at school.

## Discussion

This study determined the level of awareness and knowledge of chlamydia among young people who were approached in a variety of community settings and offered opportunistic screening. The survey data revealed a high awareness of chlamydia, which contrasts with other survey work, which reports levels of awareness of chlamydia in men and women to be around 50-60% [30-34]. These studies were with clinical populations but poor knowledge has also been reported among non-clinical populations, including medical students, other university students and school pupils, [35-37]. Nevertheless, whilst awareness was high, knowledge decreased as questions became increasingly focussed, so that the majority of respondents knew they could not catch chlamydia from toilet seats but few knew chlamydia could cause conjunctivitis.

The participant observation data, specifically the recorded observations made during the initial approach to young people, revealed gender differences in the degree of certainty of having heard of chlamydia: women immediately reported having heard of chlamydia; in contrast, men were unsure and required prompting that it was a STI before recognising it and confirming awareness. This lack of recognition among men could have implications for social marketing approaches, as men may not instantly recognise chlamydia as an STI or consider it to be personally relevant. These gender differences could have implications for how young people are approached and offered chlamydia screening. However, conveying information will involve more than information provision as stigma and embarrassment may reduce participation in screening [26].

Gender differences in awareness extended to knowledge of symptoms of chlamydia. The survey revealed that a third of men and women were unaware of the asymptomatic nature of chlamydia infection. Almost half of men agreed with the statement 'I'd only be concerned about chlamydia if I had symptoms'. Poor knowledge could lead to increased risk behaviour [38], and negatively impact screening uptake as young people who are better able to assess their own personal risk are more likely to take up a screening offer [18]. However, as reported elsewhere, [26] learning of the asymptomatic nature of chlamydia was central to young people's willingness to be screened in this study, which reveals the modifiable nature of their knowledge and the importance of this for subsequent participation. Others have noted the tendency for those who develop minor symptoms to delay care seeking [39], and poor knowledge of the potential implications of chlamydia infection for fertility [34], which places greater importance on ensuring that young people are aware of key aspects of chlamydia infection. Participation in screening and uptake of repeat testing are vital for early detection and treatment of asymptomatic infections [11,40].

This Scottish study population is not exposed to chlamydia screening as part of a national screening programme, thus our data are not necessarily generalisable to other populations. Nevertheless opportunistic screening guidelines in Scotland (SIGN Guideline 109) recommend screening as part of routine care in the same clinical settings as the NCSP in England: community contraception services, general practice, community pharmacies and termination of pregnancy services [4]. Despite the NCSP in England, poor knowledge of the issue is still present [25,35].

There are other limitations to this study. We used a convenience sample for the survey, and it was difficult to undertake purposive sampling for the qualitative study. Despite the care taken by the researcher to ensure a consistent approach to all age-eligible users it is possible that there was selection bias. In addition, recall and social desirability biases may have been introduced, with respondents perhaps selecting firm responses in the presence of their peers rather than answer 'Don't know'. There could also have been conferring between participants when completing their questionnaires. It is possible that responses to some questionnaire items could have been influenced by the information contained within the study literature (leaflets and posters) as well as the nature of the consent procedure - for example, the leaflet detailed female symptoms and the questionnaire asked for knowledge of female symptoms. A questionnaire-based study which does not provide such prior information about chlamydia could introduce less bias to findings. These issues could bias the study and the results should be interpreted in light of this. We did not ask participants for information on their level of education, so we do not know how far this might have affected responses to questionnaire items on knowledge. However, as noted above, poor knowledge has been reported among young people recruited across a range of settings [35-37], and this study found little setting-effect on knowledge of symptoms. A strength is the qualitative data confirming the questionnaire responses, including the gender differences in knowledge. Rigorous recording of the outcome of each approach made to young people was included in fieldnote diaries, which revealed a high participation rate. The sample size and variety of community settings used are also strengths.

Whilst the inclusion of men in screening has been widely encouraged [41-43], this study shows that their knowledge continues to be lower than that of women's, with little changing from earlier studies. Better ways to inform young men of the key features of the infection are vital if screening rates among men are to improve. The implications of poor knowledge for disease control are significant unless there is an improvement in young people's understanding of this infection. To improve chlamydia detection and treatment innovative strategies have been developed, including postal testing kits either mailed proactively from general practice lists or made available on the Internet for young people to request a kit and/or log-on to receive a diagnosis [29,44,45]. Despite these innovations, uptake remains poor. In order to maximise gains from interventions to improve detection and treatment, interventions are also required to increase young people's understanding of chlamydia, their assessment of personal risk and to change health behaviours.

## Conclusions

Communicating messages about the health risks associated with chlamydia to young people represents a public health challenge, particularly given the sexual nature of its transmission, their lack of knowledge, and its high prevalence in the target population. Greater efforts are required to translate evidence into action, and to close the gap between lay and professional knowledge regarding chlamydia infection, its detection and treatment.

## Competing interests

The authors declare that they have no competing interests.

## Authors' contributions

GH had the original idea for the study and designed the study with KL and was involved in the drafting of the paper; KL collected and analysed the data and wrote the first draft of the paper. Both authors approved the final version of the manuscript.

## Pre-publication history

The pre-publication history for this paper can be accessed here:

http://www.biomedcentral.com/1471-2458/10/794/prepub

## Supplementary Material

Additional file 1 **study leaflet**. study leaflet given to potential participants when first approached. Click here for file

Additional file 2 **Questionnaire**. self-complete study questionnaire. Click here for file

Additional file 3 **topic guide.rtf**. topic guide used during the in-depth interviews. Click here for file
