# Supplementary material for: Knowledge of Chlamydia trachomatis among men and women approached to participate in community-based screening, Scotland, UK
Source: BMC Public Health. 2010 Dec 30;10:794. doi: 10.1186/1471-2458-10-794 (PMC3022863; doi:10.1186/1471-2458-10-794)
Supplement: Additional file 1 — study leaflet. study leaflet given to potential participants when first approached. [file 1471-2458-10-794-S1.PDF]

## What should I do if my partner or I have symptoms and may be infected?

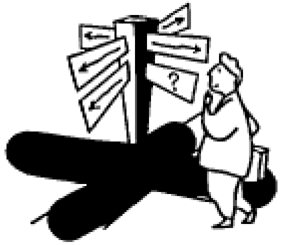

You can be tested with this study and if positive you will be referred for correct treatment. However, if you are concerned about symptoms and wish to seek advice on your own you can attend your doctor or nearest genitourinary medicine (GUM) clinic.

## How is Chlamydia tested for?

Both women and men can be tested for Chlamydia with a urine sample. Sometimes a swab might be taken, but this is increasingly being replaced with the urine sample.

Whilst it might be a bit embarrassing at least you do it in private on your own - no one else is there with you and it can easily be done in the toilet. It is quick and painless.

The samples are sent to a laboratory to be tested. In this study, they will not have your name on it.

## What if I have Chlamydia?

You will be referred for treatment with antibiotics. It is important that if you receive a positive result for Chlamydia that you do seek treatment and also tell your current/previous partner(s) as they will also need to be tested. You will not have to contact them if you do not wish to; you can receive assistance from GUM staff.

*If you have a partner* you will have to abstain from sex until a week after you have been given your course of antibiotics.

You may become re-infected if you do not follow treatment.

**Useful contacts:**
**Sandyford Initiative**
**2 – 6 Sandyford Place**
**(near Charing Cross)**
**Tel: 0141 211 8601**
**Drop-in service, with some appointments**

## My contact details:


Karen Lorimer
University of Glasgow
1 Lilybank Gardens, Glasgow, G12 8RZ
Tel: 0141 330 3295 (Direct Line)
Email: k.lorimer@clinmed.gla.ac.uk


**UNIVERSITY**
*of*
**GLASGOW**

# Chlamydia...

## ...the 1 in 10 bug. What is it? Should I be tested?

## What is Chlamydia?

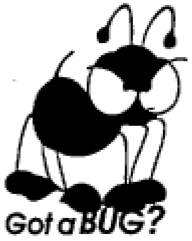


*Chlamydia* is a bacterial sexually transmitted infection (STI). It is the most common sexual infection in the UK.

## How do you get it?

Chlamydia is mostly sexually transmitted. You can catch it by having unprotected sex with someone who has Chlamydia.

(*on rare occasions*, the infection can be passed on the fingers from the genitals to the eyes causing conjunctivitis and a woman with a genital infection can pass it to her baby during birth).

## Can it be cured?

Yes, Chlamydia can be easily treated with a short course of antibiotics.

## Can it be prevented?

Using condoms during sex can reduce the risk of catching Chlamydia, as well as other STIs.

## But, if my sex partner has no obvious signs of an STI, won't I be OK?

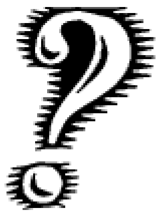

Around 80% of women and 50% of men with Chlamydia will have **no symptoms**. So, they will have the infection and not know about it. They may then pass it on to you and you may not have symptoms either and not know you have an infection.

For those *with symptoms* they might include:

**IN WOMEN:**

- An unusual vaginal discharge
- The need to pass urine more often
- Pain on passing urine or during sex
- Bleeding between periods or after sex.

**IN MEN:**

- A discharge from the penis
- Pain and/or burning when passing urine
- Irritation at the tip of the penis

## What is the risk of getting infected?

Chlamydia is extremely common, especially among those under 25. It's estimated that around **1 in 10 people are infected.** In the UK there are over 60,000 *reported* cases of Chlamydia. It is estimated that only around 10% of infections are actually diagnosed, so there's a large pool of people in the population who do not know they have chlamydia.

## If it can be treated then what's the problem?

It can be carried for a long time. It is thought that Chlamydia can go on to cause quite serious problems such as:

**In women**

*Pelvic Inflammatory Disease* (PID) that can be very painful and take months to treat;
Damage to the fallopian tubes (the tubes that connect a woman's ovaries with her womb) causing a fertilised egg to get stuck and resulting in an *ectopic pregnancy*.

**In men**

Inflammation of the testicles (epididymitis)
Fertility problems
