# Supplementary material for: Knowledge of Chlamydia trachomatis among men and women approached to participate in community-based screening, Scotland, UK
Source: BMC Public Health. 2010 Dec 30;10:794. doi: 10.1186/1471-2458-10-794 (PMC3022863; doi:10.1186/1471-2458-10-794)
Supplement: Additional file 2 — Questionnaire. self-complete study questionnaire. [file 1471-2458-10-794-S2.PDF]

UNIVERSITY
of
GLASGOW

Chlamydia Screening Study

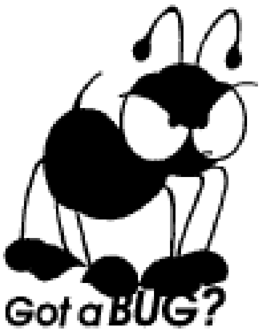

What do young people aged 16 to 24 think about testing for chlamydia in non-medical settings.

For Office Use Only:

Study No. ☐☐☐

Location: ☐☐☐

Date: ☐☐☐☐☐☐

Part A: About chlamydia...

IMPORTANT: If you haven't had sex, please attempt questions 1—16 and question 21. Thanks!

1 **Had you heard of chlamydia before this study?**

- Yes ☐
- No ☐

2 **What is chlamydia?** **(Tick one)**

- A blood disorder ☐
- A pregnancy complication ☐
- A vitamin ☐
- A sexual infection ☐
- A form of contraception ☐

3 **Where did you first hear about chlamydia?** **(Tick one)**

- This study ☐
- Friend ☐
- Family member ☐
- Doctor/nurse ☐
- Teacher (school) ☐
- Internet ☐
- Magazine ☐
- Television ☐
- Health information leaflet ☐
- Other *(please state)* ____________________

4 **How do you rate your knowledge about the following:**

	Excellent	Good	Fair	Poor
How you get chlamydia	☐	☐	☐	☐
Symptoms of chlamydia in women	☐	☐	☐	☐
Symptoms of chlamydia in men	☐	☐	☐	☐
Medical problems that having chlamydia long-term can cause	☐	☐	☐	☐
How to test someone for chlamydia	☐	☐	☐	☐

5a **How can you get chlamydia?** **(Tick one)**

- Sharing cups ☐
- Kissing ☐
- Toilet seats ☐
- Dirty needles ☐
- Unsafe sex (no condom) ☐
- Other (*Please state*) ____________________

5b **For those WOMEN who develop symptoms, what might they be?** **(You can tick more than one)**

- Unusual discharge ☐
- Pain or stinging when peeing ☐
- Dizziness ☐
- Headache ☐
- Pain during sex ☐
- Pain in lower stomach ☐
- Itch and/or rash ☐
- *Don't know* ☐

5c For those MEN who develop symptoms, what might they be? (You can tick more than one)

- Unusual discharge from tip of penis ☐
- Pain and/or burning when peeing ☐
- Dizziness ☐
- Headache ☐
- Pain/swelling in testicles ☐
- Itchiness around groin ☐
- Rash ☐
- *Don't know* ☐

5d **Chlamydia may cause problems in women who have it for a long time.**

Do you know what problems? Please state: ______________________

____

____

Don't know ☐

5e **Chlamydia may cause problems in men who have it for a long time.**

Do you know what problems? Please state: ______________________

____

____

Don't know ☐

5f How would you be tested for chlamydia? **(Tick one)**

- Saliva sample ☐
- Blood sample ☐
- Urine sample ☐

6 **True or False?**

	True	False	Not sure
• You can catch chlamydia from toilet seats..................................	☐	☐	☐
• Men with chlamydia **might not** have symptoms........................	☐	☐	☐
• Most women will not develop symptoms of chlamydia......	☐	☐	☐
• Only women get chlamydia...	☐	☐	☐
• Chlamydia can affect men's fertility...	☐	☐	☐
• Chlamydia can affect women's fertility..	☐	☐	☐
• Chlamydia can cause eye infections (conjunctivitis)...............	☐	☐	☐
• Once you get chlamydia you can't get rid of it..........................	☐	☐	☐
• You can get chlamydia more than once...	☐	☐	☐
• Women's smear tests would detect chlamydia............................	☐	☐	☐
• 'The Pill' prevents sexual infections..	☐	☐	☐

Part B: Testing

7a When you were approached and asked to take part in a chlamydia study, did you feel embarrassed?

- Not at all embarrassed ☐
- A little embarrassed ☐
- Yes, very embarrassed ☐

7b If you were embarrassed, WHY?

- I get embarrassed quite easy ☐
- I was with people I don't know well ☐
- People were looking at me ☐
- I didn't know anything about Chlamydia ☐
- Other (Please state) ______________________

8 When you were approached and asked to take part in a chlamydia study, how willing were you to take part?

- Very willing ☐
- Fairly willing ☐
- Not very willing ☐

9 You were asked to take part in this research by a woman. Would you have *preferred* to be asked by a man? (Tick one)

Yes	No	No preference	Not sure
☐	☐	☐	☐

10a **Are other people taking part in this research with you?**

Yes ☐

No, I'm by myself just now ☐ —— **Go to question 11**

10b **<u>If YES</u> to the last question:**

Do you think you would only consider being tested for chlamydia if they would too?

Yes	No	Maybe	Not sure
☐	☐	☐	☐

11 <u>Thinking about being asked to provide a urine sample in work</u> :

How far would the following <u>influence</u> you to give a sample

	Strongly Influence	Influence a little	Not Sure	Not really influence	Strongly <u>not</u> influence
Location of the toilet (in which to give a sample)	☐	☐	☐	☐	☐
How many people are around you at the time you are asked	☐	☐	☐	☐	☐
Whether you could be bothered to give a sample at the time	☐	☐	☐	☐	☐
How much you feel you know about chlamydia	☐	☐	☐	☐	☐
How much time you have when you are asked to give a sample	☐	☐	☐	☐	☐

Is there anything else not mentioned that **<u>would</u>** influence you? ____________________

B IF YOU WERE TESTED FOR CHLAMYDIA, would you be worried that your sample would be tested for things other than chlamydia (eg, drugs...)?

- Yes ☐
- No ☐
- Not sure ☐

C Would you be embarrassed to give a urine sample in work?

- Yes, a lot ☐
- Yes, a little ☐
- Not at all ☐
- Don't know ☐

12 Do you consider yourself at risk of having chlamydia?

- Yes ☐
- No ☐
- Maybe ☐
- Not sure ☐

13 Have you ever been tested for chlamydia infection?

- Yes ☐ —— If yes, when MTH ☐ YEAR ☐
- No ☐
- Not sure ☐

NB. Chlamydia is tested by providing a sample of urine. This can be done anywhere there is a toilet facility.

14 **How acceptable <u>to you personally</u> would it be to test for chlamydia infection in the following settings?**

	Very Acceptable	Acceptable	Not very Acceptable	Not Acceptable
Doctors	☐	☐	☐	☐
Family planning clinic	☐	☐	☐	☐
Termination of pregnancy clinic (abortion)	☐	☐	☐	☐
Boots (chemist) or Superdrugs	☐	☐	☐	☐
Local pharmacies	☐	☐	☐	☐
Health & Fitness (e.g. gyms)	☐	☐	☐	☐
Internet cafes	☐	☐	☐	☐
High school	☐	☐	☐	☐
College/University	☐	☐	☐	☐
Pubs	☐	☐	☐	☐
Clubs	☐	☐	☐	☐
Workplace	☐	☐	☐	☐
Prison	☐	☐	☐	☐
Army barracks	☐	☐	☐	☐
Bookstores (e.g. Borders...)	☐	☐	☐	☐

15 **How far do you agree/disagree with the following statements?**

(Please *tick* ***one*** *answer* for each statement)

PERSONAL:

	Strongly Agree	Agree	Not Sure	Disagree	Strongly Disagree
• I'd know if I had chlamydia...............................	☐	☐	☐	☐	☐
• I would worry about pregnancy more than sexual infections........................	☐	☐	☐	☐	☐
• I'd only think about chlamydia if I had symptoms..	☐	☐	☐	☐	☐
• I'd feel embarrassed going to a clinic to be tested for a sexual infection...........................	☐	☐	☐	☐	☐
• If I caught a sexual infection it wouldn't bother me..	☐	☐	☐	☐	☐

GENERAL:

	Strongly Agree	Agree	Not Sure	Disagree	Strongly Disagree
• Men should be tested for chlamydia............	☐	☐	☐	☐	☐
• Sexual health is more of a woman's concern than a man's..	☐	☐	☐	☐	☐
• Only doctors (medically trained persons) should test people for sexual infections......	☐	☐	☐	☐	☐

Part C: Sexual Lifestyle

The following questions ask about your sexual experience. They are important to young people's risk of chlamydia (e.g. condom use and sexual partners) *All your answers are IMPORTANT.* Please try to be as honest as you Thanks!

16

Have you ever had sexual intercourse? Yes ☐

(n.b. Only include penetrative vaginal sex). No ☐ Go to question **21**

17a Altogether, in your life so far, how many people have you had sex with
(n.b. Penetrative vaginal sex—don't include anal or oral)

Write the number in your life (so far) ☐

17b Are you certain of that number?

Certain ☐

I've estimated it ☐

18a How many people have you had sex with in the last 6 months?

Write the number ☐ —— If none write 0

18b Thinking about your three most recent sexual partners, please state your condom use with each:

	1. Most recent sexual partner	2. One before that	3. One before that
Yes, always	☐	☐	☐
Most of the time	☐	☐	☐
Some of the time	☐	☐	☐
Rarely	☐	☐	☐
Never	☐	☐	☐

19a **How old were you when you first had sex?**

Write age in years ☐

19b **Did you use a condom this first time?**

Yes ☐

No ☐

19c The person you had sex with **MOST RECENTLY**, are you now or were you ever...

Married to each other ☐
Or, living together as a couple ☐
Or, regular partners but never lived together ☐
Or, not regular partners (so far) ☐

20a Next to each infection, tick the box if a doctor (or other medically trained) person has ever told you that **you had** that infection:

Genital warts ☐
Genital herpes ☐
Gonorrhoea ☐
Chlamydia ☐
Hepatitis ☐
HIV ☐
Yes, can't remember which ☐
NO, NONE ☐ —— **Go to question 21**
OTHER (please state) ________________

20b **If YES to any:**

Where were you **tested** for the infection? ________________

21 **How old are you?**

Write age in years ☐

NB. Recent statistics:

- Chlamydia infection has increase by **300%** in the last 5 years in Scotland;
- Approx. **1 in 10** sexually active people under 25 have chlamydia;
- Around **80%** of women and **50%** of men who have chlamydia **do not know they have it;**
- It's easy treated with a short course of antibiotics.

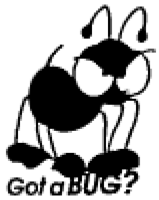

As part of this study, you can be tested for chlamydia here *today*.

All you need to do is:

- Provide a urine sample
- Give contact details (e.g. mobile phone no.)
- Within a week you will be contacted with your result (e.g. by text message).

ONLY YOU will be told your result.

ALL results are CONFIDENTIAL

22 ***Are you willing to give a sample today to be tested for chlamydia?***

Yes ☐ → Please tell Karen (the researcher) you would like to be tested.

Not sure ☐ → Ask Karen for more info. if not sure.

No ☐

If NO, it would be really useful to know why not?

____

____

END OF QUESTIONNAIRE

THANK YOU!!
