# Supplementary material for: Knowledge of Chlamydia trachomatis among men and women approached to participate in community-based screening, Scotland, UK
Source: BMC Public Health. 2010 Dec 30;10:794. doi: 10.1186/1471-2458-10-794 (PMC3022863; doi:10.1186/1471-2458-10-794)
Supplement: Additional file 3 — topic guide.rtf. topic guide used during the in-depth interviews. [file 1471-2458-10-794-S3.RTF]

Appendix 1:  Interview Schedule (without prompts)

General introduction:  related to use of setting (how long at college and what doing; how long working here..)

When did you first become aware of the study taking place in [setting name]?

What did you think about there being a study about chlamydia in [setting name]?

Were you aware of chlamydia before the study started in [setting name]?

How did you feel being approached in [setting name] and asked to take part in a study about chlamydia?

Tell me what you know about chlamydia.

(Show list of media)  Can you have a look at this list of various media and think whether you remember hearing or reading about chlamydia from any?  Tell me what you remember.

As part of the study you were offered a test for chlamydia.  How did you feel about being offered a test for chlamydia at college/the gym/your work?

Some people think asking questions about health should only be done in medical locations.  What's your view about that?

Is there anything you think you wouldn't want to talk about in this setting?

(Show list of settings – mix of medical and non-medical)  Which of these settings, in your opinion, are suitable places in which to offer chlamydia testing? (and why?)

Have you ever been tested for a sexually transmitted infection before?

Sexual history:  current/most recent sexual relationship, first sexual relationship, then summary of each in between (focusing on condom use and type of partnership (casual…))

Have you ever worried about STIs (is it something that has ever concerned you)?
